# Deliberate and emergent strategies for implementing person-centred care: a qualitative interview study with researchers, professionals and patients

**DOI:** 10.1186/s12913-017-2470-2

**Published:** 2017-08-04

**Authors:** Öncel Naldemirci, Axel Wolf, Mark Elam, Doris Lydahl, Lucy Moore, Nicky Britten

**Affiliations:** 1 Department of Sociology and Work Science, University of Gothenburg, Gothenburg, Sweden; 2 Institute of Health Care Sciences, Sahlgrenska Academy, University of Gothenburg, Gothenburg, Sweden; 3 Gothenburg Centre for Person Centred Care (GPCC), University of Gothenburg, Gothenburg, Sweden; 4 Institute of Health Research, University of Exeter Medical School, Exeter, UK

**Keywords:** Person-centred care, Implementation strategies, Normalization process theory, Deliberate and emergent strategies, Qualitative

## Abstract

**Background:**

The introduction of innovative models of healthcare does not necessarily mean that they become embedded in everyday clinical practice. This study has two aims: first, to analyse deliberate and emergent strategies adopted by healthcare professionals to overcome barriers to normalization of a specific framework of person-centred care (PCC); and secondly, to explore how the recipients of PCC understand these strategies.

**Methods:**

This paper is based on a qualitative study of the implementation of PCC in a Swedish context. It draws on semi-structured interviews with 18 researchers and 17 practitioners who adopted a model of PCC on four different wards and 20 patients who were cared for in one of these wards. Data from these interviews were first coded inductively and emerging themes are analysed in relation to normalization process theory (NPT).

**Results:**

In addition to deliberate strategies, we identify emergent strategies to normalize PCC by (i) creating and sustaining coherence in small but continuously communicating groups (ii) interpreting PCC flexibly when it meets specific local situations and (iii) enforcing teamwork between professional groups. These strategies resulted in patients perceiving PCC as bringing about (i) a sense of ease (ii) appreciation of inter-professional congruity (ii) non-hierarchical communication.

**Conclusion:**

NPT is useful to identify and analyse deliberate and emergent strategies relating to mechanisms of normalization. Emergent strategies should be interpreted not as trivial solutions to problems in implementation, but as a possible repertoire of tools, practices and skills developed in situ. As professionals and patients may have different understandings of implementation, it is also crucial to include patients’ perceptions to evaluate outcomes.

## Background

As ‘deliberately initiated attempts to introduce new, or modify existing patterns of collective action’ [1], complex interventions may be ostensibly adopted by practitioners, but they do not necessarily become embedded in everyday clinical practice. The study of implementation strategies as “methods or techniques used to enhance the adoption, implementation, and sustainability of a clinical program or practice” ([2], p.2) has gained salience in the literature. As these strategies have been rarely defined [3], there have been significant attempts to avoid inconsistent language use and inadequate descriptions [3–6] and to clarify models and theories used in implementation [7]. As Greenhalgh et al. [8] argue, implementation is often a nonlinear process characterized by multiple shocks, setbacks, and unanticipated events ([8], p. 610). Local adaptations are inevitable [9]. In order to change the way healthcare is delivered, it is imperative to take into account the present structures, attitudes and assumptions and how potential and actual challenges are handled by implementers and early adopters. At this point, we want to draw upon deliberate and emergent strategies as they were conceptualised by Mintzberg and Water [10] not to add to the taxonomy of *types* of strategies, but rather to highlight the *nature* of implementation strategies. Deliberate strategies are strategies that are introduced as intended according to a specific agenda set by an organisation, while emergent strategies arise in response to contingencies encountered when pursuing deliberate strategies. In other words, deliberate strategies appear prior to the initiation of a novel type of collective action and offer prescriptions and itineraries based on evidence and intuition (see also [5]), whereas emergent strategies imply “learning what works–taking one action at a time in search for that viable pattern or consistency” ([10], p.271). By employing this classification, we want to emphasise that challenges to implementing a complex intervention are collectively and creatively interpreted and handled in practice [11]. Rather than driving a wedge between ‘fidelity’ and ‘flexibility’, this classification may help to explain the dynamic, often corrective and adaptive nature of implementation strategies. For complex interventions, there are often planned responses to expected problems. It is crucial to see how a set of practices are locally interpreted and modified in practice [12], which is often possible due to emergent strategies.

Over the past half century paternalistic and disease-focused approaches in healthcare have been criticized. There is a growing literature on patient-centred consultations [13], patient-centredness [14, 15] and person-centred care [16–19] to underline care as “respectful and responsive to individual patient preferences, needs, and values” ([20], p.49). Even though the terms ‘patient’, ‘client’ and ‘person’ are often used interchangeably in the literature [21], the idea of attending to the person behind the patient has paved the way for interventions in terms of enabling and encouraging persons to bring their knowledge, preferences and capabilities into decision making and care planning [19]. In this way, person-centredness acknowledges to a higher degree compared to patient-centredness the resources and capacities of the person. The study of strategies for implementing these relatively new models of patient and person-centred care is crucial for better understanding the changes they aim for in preparing, enacting and delivering healthcare.

A recent evidence-based person-centred care (PCC) framework developed by the University of Gothenburg Centre for Person-Centred Care (GPCC) was implemented in different healthcare contexts in Sweden [22, 23]. This framework involves three practices that are intentionally called ‘routines’; collecting the patient’s narrative, establishing partnership by setting goals together and documenting this partnership [22, 24]. These routines require behavioural, technological and organisational changes; hence the model fits in the definition of a complex intervention [1]. The choice of the term ‘routines’ implies that implementation is conceived to embed and sustain certain everyday practices. The GPCC framework has been shown to be effective in clinical studies for people with hip fractures [25, 26], chronic health failure [27], rheumatoid arthritis [28] and acute coronary syndrome [29]. However, the implementation of the framework has attracted less scholarly attention [30, 31]. Further research is needed to understand how the framework can be introduced and how and whether it can be sustained.

This study has two aims: first, to explore the deliberate and emergent strategies of key stakeholders to specific contextual challenges encountered when implementing the GPCC framework; and secondly, to explore how the recipients of PCC perceived the effects of these strategies.

## Methods

### Sample and design

Qualitative semi-structured interviews were conducted with three groups. We used a purposive sampling strategy to select researchers and healthcare professionals and a convenience sampling approach for patients. The first group of respondents (*n* = 18) were researchers in different PCC intervention studies selected to represent different contexts of PCC (see also [11, 24, 32]). The second group (*n* = 17) were healthcare practitioners who worked in hospital wards enrolled in a larger PCC implementation project at a university hospital in Sweden, or in other healthcare settings used as study sites for GPCC interventions. Hospital wards varied in size, specialisation and patient group. Practitioners took part in a 10-week PCC change management programme, dealing with the ethics of PCC and results from PCC studies. This includes training in developing tools such as care plans and interview techniques. Each ward manager was contacted with information about the study and gave their consent. They were asked to recruit a nurse, an assistant nurse and a physician with experience of working with PCC. Five registered nurses, four assistant nurses, four ward managers and four physicians participated in the study. The third group (*n* = 20) were patients who had recently been hospitalised, at the time of the interview, to a medical ward where the GPCC framework had been systematically implemented over several years. Patients were eligible for enrolment into the study if they were cognisant and able to communicate in Swedish. A nurse coordinator provided eligible patients with the study information (see also [33]).

### Data collection

Interviews with researchers were conducted by AW, DL, ME, ÖN. Interviews with practitioners were conducted by AW, DL and a research assistant (MH) and the interviews with patients were conducted by MH. Slightly different topic guides were used for the three different groups (see these guides in [33]). Questions for researchers and practitioners sought to elicit how PCC related to the everyday work of healthcare practitioners. An additional topic guide was used for the patient interviews [33]. This topic guide was intended to elicit patients’ experiences of care on the ward and their understanding of PCC. Guides were not restricted to questions concerning the implementation of PCC; they rather sought to uncover a wide range of topics about the philosophy, practice and definition of PCC. Interviews lasted between 29 min and 1 h and were audio recorded, transcribed verbatim, anonymised and translated from Swedish into English. Interviews with patients were conducted in their homes, on the hospital ward or by telephone according to their preference. All interviewees were given a pseudonym to maintain anonymity (Researchers represented with R, healthcare practitioners with H and patients with P). All participants prior to their interview were provided written and verbal information and gave informed consent. The study was approved by the regional ethics committee in Gothenburg.

### Data analysis

Each transcript was first coded inductively. For the interviews with researchers and practitioners, descriptive codes were created to map different challenges to implementation and the strategies reported by the interviewees. After the initial coding, strategies to overcome reported challenges were divided into two groups as deliberate and emergent [10]. Then, using the framework method [34], challenges to implementation, deliberate and emergent strategies were grouped and analysed according to four components of Normalization Process Theory [35, 36]. NPT is an influential framework used to understand and assess the implementation of complex interventions in healthcare, especially technological interventions, and has been shown to be useful in several studies [37–40]. Normalization means that an intervention becomes ‘routinely embedded in the matrices of already existing, socially patterned, knowledge and practices’ [35]. This sets normalization apart from a simple adoption or insertion of a new set of ideas and practices into an organisational vacuum.

NPT suggests four generative mechanisms of normalization: coherence (the sense-making work), cognitive participation (relational work), collective action (operational work) and reflexive monitoring (appraisal work) [35, 36]. Coherence is about how people make sense of the intervention, how they individually and collectively comprehend what is at stake. Cognitive participation is about how people engage in relationships with others in order to initiate and fulfil the requirements of a course of action. Collective action is about enacting, thereby making the intervention part of daily practice. Reflexive monitoring is the evaluation of what is being done in daily practice and how the intervention changes or fails to alter daily practice. Each mechanism is subject to challenges depending on the nature of the intervention, how and where it is introduced, and by whom. These NPT constructs helped analyse different challenges and strategies in a systematic fashion (see Table 1). Interviews with patients were coded inductively to delineate some common themes about the ways they perceive implementation and operationalisation of PCC.

**Table 1:** Mapping of GPCC challenges and strategies against NPT constructs

Constructs of NPT	Challenges	Deliberate strategies	Emergent strategies
Coherence (sense-making work) (1) Differentiation (2) Communal specification (3) Individual specification (4) Internalization	Conflicting and/or divergent views and expectations about PCC Translating abstract principles into concrete practices	Education and seminars organized by GPCC Design of the training programme allowing for contextual adaptations and developments	Lunch seminars, informal meetings, inter-professional discussions in small groups Invited lectures and seminars Individual and/or collective ways of relating to professional experiences
Cognitive participation (relational work) (1) Initiation (2) Enrolment (3) Legitimation (4) Activation	Resistance (to change) Force of habit Different approaches among professionals (mainly nurses and doctors) Fatigue from previous implementations	Education and seminars organized by GPCC Use of research-based evidence (e.g. reduced time of hospitalisation)	Using leading personalities, initiators (“ambassadors”), engaging previous personal relations at work Convincing and motivating unwilling actors (e.g. doctors with “scientific” evidence) Interpretation and collective development of routines (e.g. documentation)
Collective action (operational work) (1) Interactional workability (2) Relational integration (3) Skill set workability (4) Contextual integration	Time shortage Organizational problems (rotation of the staff and the physical environment) Inter-professional hierarchies (mainly between nurses and doctors) Different patient groups with specific conditions and needs Division of workload (e.g. increased documentation)	Funding and extra staff (research nurses) Initiating teamwork Transfer of expertise via researchers and experienced implementers Use of scales and technologies	Commitment and support of managers (e.g. initiating and consolidating teamwork) Strengthening teamwork by engaging all expertise in the team (the patient included) and empowering nurses to contribute more to decision making Developing new practices to safeguard continuity (e.g. introduction programmes for new staff
Reflexive monitoring (appraisal work) (1) Systemization (2) Communal appraisal (3) Individual appraisal (4) Reconfiguration	Time shortage Increased number of patients and more workload from documentation	Focus on shortening hospitalisation time	Small group discussions, “ethical forums” Continuous education, evaluation in practice Evaluation of the workload

## Results

First, we will present the barriers to the four mechanisms of normalization, and deliberate and emergent strategies responding to these barriers as reported by researchers and practitioners (see Table 1). In the second part, we will explain three effects of these strategies on patients: they felt a sense of ease and reported inter-professional congruity and non-hierarchical communication.

### Barriers and emergent strategies

#### Ensuring coherence: Small-group and informal meetings

One deliberate strategy was to introduce PCC by organizing structured and expert-facilitated change management programmes for healthcare practitioners. Yet, the curriculum was open to interpretation and the training was not designed to impose clear-cut answers to context-specific needs and practices. As one senior researcher suggested:

There is obviously the definition we use in GPCC [it] is not, does not need to be the same for everyone. There could be other, and there are other ways of viewing this where you set up other criteria. (R7)

Interviewees reported two main challenges to the coherence of the framework: conflicting and/or divergent views about PCC and the difficulty of translating abstract principles into concrete practices. As there have been previous approaches addressing disease-focused attitudes in healthcare since the 1960s, some interviewees pointed out the continuities between different holistic care models and PCC:

There’s always been person-centred healthcare, but we’ve called it different things throughout the course of history: humanistic nursing and all the possible different angles of approach. (R2)

Similarly, some practitioners did not realize what PCC would change in their actual practice:

Some may feel like this is obvious and that we always work in this way. This might create a resistance, like “why should we force an open door or do these obvious things?” But it may not be obvious to everyone, and if you make it more structured it becomes more visible in a different way. (H9)

The formal training by GPCC offered participants a structured way of expressing what they already thought about:

I’ve always been interested in the patients’ own narratives, and when they had attended this education and passed it onto us, you got to put it into words what you’ve always thought about in a way. (H7)

However, not everyone could attend these courses; therefore one emergent strategy was to ensure coherence by creating new forms of discussion about PCC. Managers encouraged informal meetings in small groups:

We did have lunches together with different occupational groups; nurses, assistant nurses and doctors. At least one of each group had attended the education, so we had a little brainstorming and we simply talked about what person-centred care meant for me, what it meant for the assistant nurse and for the doctor. (…) we talk about it from different perspectives and different angles all the time. (H7)

These discussions helped practitioners differentiate PCC from their usual practice. These inter-professional communication groups enabled practitioners to bring their previous experiences and individual concerns to the sense-making work. Some practitioners could link their professional experience and individual techniques to the emphasis on the person and their narratives.

#### Endorsing cognitive participation: ‘Ambassadors’ and interpreting routines

There were several barriers to the relational work that the GPCC framework required. Some professionals, mainly doctors, resisted the implementation of PCC, pointing to organizational barriers (see also [32]):

But there has been a frustration about the structure itself, and of the common understanding about why we’re doing this. This was something you noticed when you started the projects, and the doctor’s unit expressed “How are we going to have the time for this? How can we get the resources?” (H16)

One deliberate strategy to convince unwilling and sceptical practitioners of the workability of the model was to provide them with evidence of shortened hospital stay, better continuity of care or improved diagnosis:

So many old doctors that have been here like a very long time, since the hospital was established, and they think, like ‘Well, what is this innovation? Why should we do it like this?’ And then you need to try to change them, in a way, and show them that this way, in this way we can shorten the care period and we can save in this area or the patients are happier and stuff like that. But it’s time, it all takes time. (H3)

An emergent strategy was to address the unwillingness of the staff via ‘pioneers’, whom two interviewees called ‘ambassadors’. Ambassadors were seen to mediate between established practices and PCC:

We are currently trying to dedicate two nurses to be ambassadors. (…) My thought is that they can get some time off to be out in the units to support them, to see that they are able to engage this approach and understand and understand the concept, and later on be able to measure and follow up on the number of care plans for example, or be able to interview co-workers and patients about how they have experienced this. (H16)

One reported barrier to implementation was perceived to be force of habit (see also [31]). It was difficult to change established ways of working:

All workgroups need strong leaders because there is usually a grey, there is a grey mass, which does not want, or do not want change, and they need to be pushed and we need, these strong leaders need to make available the resources and opportunities for this. It’s needed. Then I don’t think one needs anything else. (H5)

The duration of training was short and once practitioners returned to work, there was the risk of “falling back”:

So I think it needs more things than just being educated in person-centred care because you can go to the education, Ok, this is how to do it then you will go back to do work, and do like you always do. (R12)

Therefore, an emergent strategy was to interpret and develop routines simultaneously during the implementation. For instance, practitioners in different wards were spurred on to create and test different forms of documenting the patient narrative and partnership. In the GPCC framework, documentation was considered to give legitimacy and transparency to patient perspectives and partnership, and consistency to the care chain [22]. However, it was also the most challenging aspect of PCC since it was often time-consuming and incompatible with existing medical record systems [24]. Documentation also needed to vary depending on the characteristics and needs of patients who had different medical conditions and resources:

the groups who have worked with this have got together to introduce a proposal for what the care plan would be like, how you would develop it and how you would get the patient to participate in it. (H16)

I think this is the way everyone wants to work but of course you have to find your own PCC-documentation (H6)

This generated different forms of documentation on different wards. It depended mostly on the needs and characteristics of the group of patients practitioners encountered:

When it comes to certain categories of patients, this [PCC] might feel unnecessary, for example, when certain patients aren’t able to speak or might be gravely demented. When you can’t have a discussion with them and you won’t be able to include them in the planning. At times like this the team decisions might tend to get shorter, but we still write them down, and there are often relatives around with whom you can have a dialogue. (H9)

As the above quote illustrates, the clinical encounter influenced what could be documented and in which form. This also led practitioners to use a less medical vocabulary and share more information in an accessible way with patients:

The idea is that it should be written in a simple language, with simple words. I tend to write it in second-person form, which you usually don’t do in the medical record. (H9)

#### Enabling collective action: Enforcing teamwork

One barrier to collective action was the real and perceived organisational problems such as time constraints, rotation of practitioners, the increasing need for documentation and increased workload. Some practitioners lacked an understanding of the philosophy behind PCC and focused only on practices:

When everyone on the clinic was supposed to work with this [PCC], we did protest quite a lot since we felt that we never would manage to do this, that there only would be paperwork. The deeper understanding for this was definitely missing. (H13)

Another barrier was the inter-professional hierarchy between doctors and nurses. The implementation of this framework depended upon better cooperation between different professional groups. However many early adopters came from the nursing profession and some doctors were not initially keen to embrace the model. Existing hierarchies posed problems for the collective action and support of managers was required:

To be able to succeed and work person-centered you need to have everybody on board. Or at least most of them and the right people I say. Because there are always leading personalities in a ward and if they are with you it’s often a bit easier to get the rest of the staff on board too. They make it happen. But it has to start at the top. I mean, it’s the whole chain down. (H4)

As the person and their goals and wishes are at the centre of PCC, some nurses felt better placed to elicit these goals and wishes, they needed doctors to engage more in teamwork:

In person-centred care nurses get a new role, actually, in the team, because we have our individual task to perform (…). But we have a different role and we need to work together with the physician, not *under* the physician. (…) once they get the hang of it, they will like this a lot because today the physicians are responsible for *everything*, also in areas where they don’t have any training or knowledge at all. (R8)

However, existing hierarchies between professional groups were not explicitly addressed by GPCC, which rather emphasised teamwork as a particular form of collective action that should lead to an improved work environment:

Person-centred care creates a certain work environment, with a better structure of the work. It facilitates the planning for doctors and nurses, not to make late decisions but rather to have something to work with. This facilitates to make a better work environment. I also think this spreads a certain interest, that it can be exciting to be part of it from the beginning (H16)

Even though teamwork was deliberately emphasized by GPCC, it needed to be reinforced by managers who spent extra time and effort to ensure nurses and especially assistant nurses assumed their role without hesitation:

You should know that you actually add something and what you see is also important, because it’s a team. (H1)

As staff turnover was high, another difficulty was about transferring collective ways of working to new or substitute staff. GPCC did not have a clear deliberate strategy to deal with staff rotation and it was rather difficult to manage the continuity of collective action. An emergent strategy was, therefore, to develop new practices to safeguard continuity in patterns of collective action. Practitioners on one ward decided to design and implement an introductory program for new staff.

This is something that you have to keep alive, which is a challenge when you are changing the personnel. This is why we developed the introduction program, to not lose it when we change the personnel, and to introduce to everyone who comes to us to work. (H16)

#### Aspiring for reflexive monitoring

Interviewees described two main problems about evaluating their work. Many practitioners reported that there was not enough time because developing and testing new techniques, for instance PCC documentation, required time. They also felt uneasy about the shortened time of hospitalisation, which they believed led to an increased turnover of patients. Practitioners, especially nurses, spent more time on documentation and had less stamina and resources for evaluating their practice:

The disadvantage is the increased workload. We have drastically reduced the time of hospitalization, which means we get a lot more patients than our neighbouring wards, and it is the days when patients arrive or are discharged which are the rough days. (H14)

Some interviewees underlined that the GPCC framework focused inappropriately on shortening hospitalisation time and that there were no specific strategies to overcome the increased workload in the case of successful implementation nor to enable continuous reflection on action. Therefore, they emphasised the need to find time to constantly evaluate their PCC practice:

Another task is to continuously evaluate the work so that feedback can be given on how the work is turning out and what effects it has brought here on the ward, or in the clinic, where it has been introduced. (H9)

A ward manager reported one emergent strategy whereby she initiated group discussions for reflecting on PCC practice that she called “ethical forums”:

We don’t have fixed topics but rather it can be about something that’s happened that has affected the group. It is often ethical dilemmas where you can ventilate your thoughts, and I think by doing this where nothing is right or wrong you establish confidence, which also creates a need to develop these approaches. (H15)

### Receiving PCC

In this study, interviews with patients showed that they were neither explicitly informed about PCC during hospitalisation nor aware of practical changes in the admission, treatment and discharge process. Yet, patients recognised differences. It may be argued that the emergent strategies elaborated in the previous section led patients to perceive PCC as (i) creating a sense of ease (ii) heightening inter-professional congruity and (iii) promoting non-hierarchical communication.

#### Sense of ease

One common theme in interviews with patients was the sense of ease they felt on the ward where PCC had been implemented. Patients noticed changes in how they were received and the attentiveness of professionals. This welcoming and personalized approach was reflected in the way patients perceived the commitment of healthcare professionals:

I have been spending quite a lot of time at hospitals, so I think they have a *very positive atmosphere*, and it feels like one is not bothering them. It rather feels like they want to take care of you. They want one to be healthy and they want one to feel well, and I think that feels good. So I think they are very positive, yes, caring. (P12)

#### Inter-professional congruity

Teamwork encouraged professionals to take on new roles and responsibilities, thus embedding the idea that they treated each other “in a person-centred way” (R14) (see also [41]) and engendering inter-professional harmony:

Then I also thought the *personnel who worked together created a quite good atmosphere*, and that’s important. Because we get to hear that sometimes down here, that “You seem to have so much fun together.” “Yes, we have.” And many patients find that very pleasant, that we like each other as a work group. […] Nice attitude toward each other and such. Because one notices that very easily as a patient, if someone doesn’t like each other or so, like short answers or something like that. That isn’t very nice. […] Or if one notices that some doctor has an unpleasant attitude and always nags their nurses and there are sighs and so on. Then one isn’t particularly confident as a patient either. (P17)

#### Non-hierarchical communication

Congruity was facilitated and sustained by a less hierarchical communication between co-workers. Once teams were established, team members were made welcome to express and bring their knowledge and suggestions to the table, especially concerning the care plan. This generated an environment where patients also felt keen to take part in discussions, ask further questions and become informed about their medical condition and treatment.

There’s no hierarchy at all here on this ward. And they are very happy together and nice to each other too. That’s so, it reflects on the whole ward. (P12)

## Discussion

This study combines three key stakeholder groups’ perspectives on the implementation of a specific PCC framework. Drawing upon Minzberg and Water [10], we demonstrate how deliberate strategies are complemented with emergent strategies seeking to implement and normalize a complex intervention in everyday practice. There have been challenges to the implementation of this framework [31, 32]. The deliberate strategy of introducing PCC via training, seminars and financing proves to be effective in disseminating the philosophy and some techniques for PCC and paving the way for collective negotiation and sense-making within the professional groups. However, although educating and financing as two discrete strategies [6] may be necessary for the dissemination of information, they are not sufficient to guarantee positive implementation outcomes [5]. Arguably, “the characteristics of environments in which [core components] are worth replicating” ([42], p.733) should be also subject to discussion before and during the training.

Practitioners, especially doctors, were sceptical about this PCC framework in the introductory phase because they did not think it was innovative or they felt they were already person-centred in their practice. This reaction undermined the coherence of the model in the initial phase and led early adopters and managers to develop strategies to create coherence. Yet, despite the formal educational package, the main learning and developing process happened via informal meetings, where staff discussed how to practice PCC on their specific ward. Early adopters and leaders worked as “purveyors” [5] to convince unwilling actors of the workability of the framework and increase awareness of its good effects.

The fact that the framework was open to interpretation led practitioners to develop the suggested set of practices, for example, in the case of documentation. Informal meetings also contributed to the development of the framework particularly when practitioners discussed real situations [43]. Therefore, it can be argued that these meetings paved the way for ‘reflecting on action’ and ‘learning in action’ [44], promoting collective action and reflexive monitoring. Enforcing teamwork and clarifying each team member’s role were emergent strategies in the implementation of PCC, since the hierarchies between different professional cultures and groups [45], such as between doctors and nurses, had not been explicitly articulated in the framework.

Once these strategies to create coherence, cognitive participation and collective action succeeded during the implementation, practitioners reported better results of co-operation, inter-professional teamwork and communication with patients. This was also reflected in patients’ responses to PCC. Even though patients did not elaborate on the benefits of specific routines of PCC, they felt a sense of ease [46] and collegial congruity on these wards. This study has shown that the normalization of the framework was experienced by patients in terms of a sense of ease and being listened to, rather than recognized in the specific language of an active partnership between different stakeholders [33]. Patients may not always be attentive to specific PCC routines that are being implemented, yet they perceive changes in the ethics of care found in the reception, environment, attitudes and commitment to collective action.

Changing attitudes among professionals and between professionals and patients has proved to be difficult [47] and requires effort [48]. Arguably, especially for sustaining complex interventions that aiming to change behavioural and professional attitudes, there must be a systematic approach to the solutions-in-the-making as illustrated for instance with ethical forums. As practitioners made sense of the framework via continuous discussion in informal meetings, they also underlined that it was necessary to create similar platforms to evaluate their PCC practice and embed it into their daily practice. This may have helped professionals to bring their ethical considerations and subjective feelings [49] as clinicians-as-persons [41] into PCC.

This study has some limitations. It draws upon a larger study which sought to explore various definitions, practices and challenges in the GPCC framework and was not solely confined to its implementation. The study at hand seeks to narrow the focus to some challenges and strategies as reported by the researchers, practitioners and patients. Therefore, the study is interpretative and not exhaustive in systematically exploring all strategies and difficulties encountered in the implementation; it rather offers important insights about the nature of these strategies and points to some possible directions for future research about the implementation of complex interventions like PCC. Second, as the study took place in a particular institutional context, the findings may not be transferable to other settings. However, it is equally important for the objective of the study to capture strategies as they emerge in specific contexts.

## Conclusions

The present study illuminates that emergent strategies not only shed light on problems related to existing conceptions, practices and relations, but they also highlight how normalization is strongly linked with creating a willingness to learn, reflect on action and negotiate division of labour and responsibilities. There is more space for emergent strategies of collective action than deliberate strategies particularly because it is not easy to predict conflicts and tensions in action. It is therefore crucial to open up the implementation of PCC for in-depth discussion to gain a shared understanding between different stakeholders including patients.

### Practice implications

- Acknowledging PCC as a complex intervention that requires emergent strategies from within to normalize the change process.
- Extra resources and support for practitioners dealing with the practicalities of the implementation.
- Creation of formal and informal discussion groups for reflexive monitoring.
- Evaluation and documentation of emergent practices (for e.g. introduction programs for new staff or ethical forums).
- Attentive study of existing hierarchies between professional groups before the implementation.
